# Synchrony and social connection in immersive Virtual Reality

**DOI:** 10.1038/s41598-018-21765-4

**Published:** 2018-02-27

**Authors:** B. Tarr, M. Slater, E. Cohen

**Affiliations:** 1 Institute of Cognitive and Evolutionary Anthropology, University of Oxford, 64 Banbury Rd, Oxford, OX2 6PN UK; 2 Department of Experimental Psychology, University of Oxford, South Parks Rd, Oxford, OX 1 3UD UK; 3 Institució Catalana de Recerca i Estudis Avançats (ICREA), 23 Passeig de Lluís Companys, 08010 Barcelona, Spain; 4 Faculty of Psychology, University of Barcelona, 171 Passeig de la Vall d’Hebron, 08035 Barcelona, Spain; 5 Department of Computer Science, University College London, Gower Street, London, WC1E 6BT UK; 6 Wadham College, Parks Rd, Oxford, OX1 3PN UK

## Abstract

Synchronising movements in time with others can have significant positive effects on affiliative attitudes and behaviors. To explore the generalizability of synchrony effects, and to eliminate confounds of suggestion, competence and shared intention typical of standard laboratory and field experiments, we used an Immersive Virtual Reality (VR) environment. Participants, represented as virtual humans, took part in a joint movement activity with two other programmed virtual humans. The timings of the co-participant characters’ movements were covertly manipulated to achieve synchrony or non-synchrony with the focal participant. Participants in the synchrony condition reported significantly greater social closeness to their virtual co-participants than those in the non-synchrony condition. Results indicate that synchrony in joint action causes positive social effects and that these effects are robust in a VR setting. The research can potentially inform the development of VR interventions for social and psychological wellbeing.

## Introduction

Synchronizing movements in time with others can have significant positive effects on affiliative attitudes and cooperative behaviors. Compared to non-synchrony, synchrony is associated with greater liking and trust^1–4^, more generous offers in economic games^5^ and stronger perceived similarities in personality^3^. Prosocial effects of synchrony are thought to be due to co-activation of action and perception networks, which blurs the normal boundary between self and other^6^. A sense of collective fate^5^, and perception of synchronous partners or groups of individuals as a coherent unit (i.e. ‘entiativity’)^7^, have also been suggested to mediate the effect.

Research on the link between synchrony and social bonding originally built in part on an identified relationship between mimicry (i.e., making a similar movement to another individual) and positive social behaviour (e.g. self-reported rapport^8^). Mimicry improves rapport between people^9,10^ and towards avatars^11,12^, which in turn influences the amount of mimicry that people perform^13,14^, thereby causing a positive feedback loop. Synchrony, like mimicry, involves coordinated interpersonal movement, with the additional element of rhythmically matched timing, which requires the prediction of movements between co-actors. Numerous controlled experiments have demonstrated the synchrony-affiliation link in a range of activities, group sizes, contexts, ages, and cultural settings. However, this overall picture conceals important methodological limitations. Controlled synchrony and non-synchrony in the laboratory is typically achieved either via direct or implied instruction to synchronize (or not) with co-participant(s)^5,15^, and/or by providing an individual auditory and/or visual rhythmic stimulus for each participant to follow irrespective of others’ behaviors (e.g., via personal headphones) – in the synchrony condition, individual stimuli are synchronized, while in the non-synchronous condition, stimuli vary across participants^1,16–19^. Other elements of the ‘jointness’ of the activity are sometimes varied to facilitate synchrony vs. non-synchrony^15^. These methods give rise to problems of interpretation.

First, explicit instructions to act similarly versus differently to other co-participants may harbor implicit suggestions of alignment vs. antagonism that engender distinct attitudinal stances. Apparent prosocial effects of synchrony vs. non-synchrony may in fact be attributable to motivations to align vs. deviate from the actions of one’s co-participants, and thus may not be specific to synchrony. Second, effects may be attributable to social-cognitive differences associated with having joint intentions to produce synchrony together vs. absence of any explicit joint intentional or shared goal orientation. One series of studies has shown that the positive social effects of synchrony are stronger when participants are instructed to match their movements (compared, for example, to when given no explicit instruction to synchronize)^20^, suggesting that joint intentionality at least partly contributes to social effects.

Third, effects may vary according to whether participants perceive the activity as a ‘together’ or joint activity. ‘Togetherness’ need not entail a specific joint intention to synchronize, but merely a shared sense that the activity is perceived as being performed together (e.g. entailing a joint goal or mutual commitment). This may be explicit or implicit, and may have an important influence on social commitment and cooperation^21^. Therefore, even where joint intentions are not expressly manipulated in establishing synchrony vs. non-synchrony, instructional priming in the synchrony condition (e.g., “you are going to perform an activity together”) or spatial arrangement of participants (e.g., inward-facing circle) could establish feelings of mutuality conducive to cooperation. If these contextual elements are not also present in the non-synchrony condition, the role of synchrony *per se* in establishing effects becomes unclear.

Fourth, in relevant real-world social activities, such as singing, dancing, marching and drumming, steady rhythmicity tends to entail both ‘togetherness’ and temporal coordination or synchrony. The purported prosocial effects of such activities (compared with non-synchronous singing, dancing, marching, etc.) in the lab could in fact result from the violation of tacit expectations of coordination in the non-synchrony condition and/or congruence with expectation in the synchrony condition. Moreover, when a normatively ‘together’ activity, such as drumming, is individualized for laboratory control (e.g., using personal headphones), the relevance of coordination/synchrony among participants potentially diminishes, leading to weaker or null effects^16^.

Finally, the relative ease of actively synchronizing vs. not synchronizing with others potentially engenders a range of between-condition differences that could influence social effects, including perceived difficulty, competence and success. In summary, although many recent studies have shown differences in prosociality after synchrony vs. non-synchrony, the effect specifically of synchronous movement remains unclear.

To overcome these problems and to isolate the role of synchrony in bonding effects, we used a Virtual Reality (VR) first-person immersive environment in which participants, represented as virtual humans, took part in a joint movement activity with two other programmed virtual humans. In an immersive VR environment, a head-mounted display provides a computer-generated image of the virtual world. A motion capture system allows the participant to be represented by a first-person perspective virtual body whose virtual movements reflect the real body movements of the participant in real-time. The result is a sensorimotor congruence (due to visual and proprioceptive alignment) that causes the participant to have an illusion of ownership and agency over their virtual body^22^. In our study, the two other virtual humans who joined the participant’s avatar in the VR environment were not controlled by other people, but instead coded to mirror the movement of the participant to varying degrees of accuracy. The participant’s virtual body therefore joined with virtual others in an activity in which synchronization was independently and covertly manipulated. This allowed us to test the effect of synchrony vs. non-synchrony on interpersonal closeness in a VR environment while avoiding common limitations of real world laboratory experiments. In addition, the study could offer valuable insight into the limits and opportunities of VR technologies for positive social engagement in virtual embodied interaction.

We hypothesized that synchrony would be associated with greater self-reported prosociality. Two behavioral measures were also implemented to measure implicit social closeness. We predicted that participants would mimic their virtual partners more in a final ‘idle’ pose and move closer to them in the synchrony condition than the non-synchrony condition. It is well established that people tend to mimic postures of others more if they have some established rapport or a sense of closeness^23^ and physical proximity can provide an implicit behavioral measure of social closeness (see, for example, Chartrand & van Baaren’s^24^ chair measure and Tunçgenç & Cohen’s^25^ ‘island game’).

## Results

A total of 250 participants were recruited in Barcelona to achieve an approximate final target sample of 80 (based on relevant precedent^26^). The large number of exclusions was due to technical problems with the motion capture, which warped the appearance of participants’ virtual body. A final sample of 76 participants (44 females; *M* age = 22.61, SD = 4.67 years) was included for analysis. Ethics approval was obtained from the University of Oxford’s CUREC and the Bioethical Committee of the University of Barcelona, and the experiment was performed in accordance with relevant guidelines and regulations.

Analysis of participants’ self-assessed degree of synchrony (on a 1–100 scale, where 1 = *not at all* and 100 = *extremely*), revealed a significant difference in how synchronized participants felt their movements were with the other characters (Mann-Whitney U test (2-tailed): *n* = 76, *U* = 86.50 (*Z* = −6.611), *p* < 0.001, *r* = −0.758), with those in the synchrony condition scoring higher on this measure (*Median (Mdn)* = 96.00) compared to those in the non-synchrony condition (*Mdn* = 32.00). Manipulation of synchrony was further checked via independent coding (Supplementary Information 1). During each participant’s VR session, the 3D positions and rotations of the participant’s body were recorded. The position and rotation data were input into custom-made playback software which re-orientated the three virtual human bodies into a line (side-by-side) to facilitate coding. Two independent coders who were blind to the hypothesis rated whether they thought the three were moving in synchrony or not, and any disagreements (22/76) were resolved following discussion between the coders in a secondary round of coding (for coding instructions see Supplementary Information 1). The final assignments (to either synchrony or non-synchrony) were identical to the pre-established conditions (see Supplementary Information 2 for a video sample of a synchrony-coded video and Supplementary Information 3 for a non-synchrony coded sample).

There were no significant differences between movement conditions with respect to personality measures, degree of confidence in remembering the movements, or in participants’ ratings of fun, enjoyment or awkwardness felt during the virtual reality task (Supplementary Information 4, Tables S1, S5 and S6). Similarly, there were no significant differences in participants’ assessments of whether the other two characters were controlled by real people (1 = *not at all* and 100 = *very much: Mdn*_*Sync*_ = 18.00; *Mdn*_*N-sync*_ = 19.00). Participants’ perceptions that they were with other people during the virtual reality task (1 = *not at all* and 100 = *very much: Mdn*_*Sync*_ = 49.00; *Mdn*_*N-sync*_ = 41.00) did not differ significantly between conditions, nor did the ratings of how successful they felt they had been at following the movement instructions during the activity (1 = *not successful* and 100 = *very successful: Mdn*_*Sync*_ = 85.00; *Mdn*_*N-sync*_ = 80.00; Supplementary Information 4, Tables S1 and S6). There was a significant overall increase in positive affect (Repeated measures ANOVA (2-tailed): *F*(1, 69) = 6.224, *p* < 0.001, η_p_^2^ = 0.367) and decrease in negative affect (Repeated measures ANOVA (2-tailed): *n* = 72, *F*(1, 69) = 14.982, *p* < 0.001, η_p_^2^ = 0.178, note four participants did not answer all the questions) following the VR activity (compared to before), but these changes did not interact with movement condition (Supplementary Information 4, Tables S1 and S7).

There was a significant difference between conditions in participants’ ratings of how difficult they found the movement task (1 = *not at all* and 100 = *very much;* Mann-Whitney U test (2-tailed): *n* = 76, *U* = 419.00 (*Z* = −3.186), *p* = .001, *r* = −0.365), with those in the synchrony condition reporting that it was more difficult (*Mdn* = 4.00) than those in the non-synchrony condition (*Mdn* = 1.00). Although no significant difference was found between conditions in participants’ assessment of how often they were following the other characters’ movements (1 = *never* and 100 = *always*), those in the synchrony condition reported feeling that they were being followed more of the time (*Mdn* = 98.00) compared to those in the non-synchrony condition (*Mdn* = 55.00; Mann-Whitney U test (2-tailed): *n* = 76, *U* = 205.50 (*Z* = −5.3880), *p* < 0.000, *r* = −0.615). Participants’ ratings of difficulty and assessments of how often they felt they were being followed by the other characters were included as covariates in subsequent parametric analyses of the dependent variables.

### Self-report social closeness

**Figure 1:**
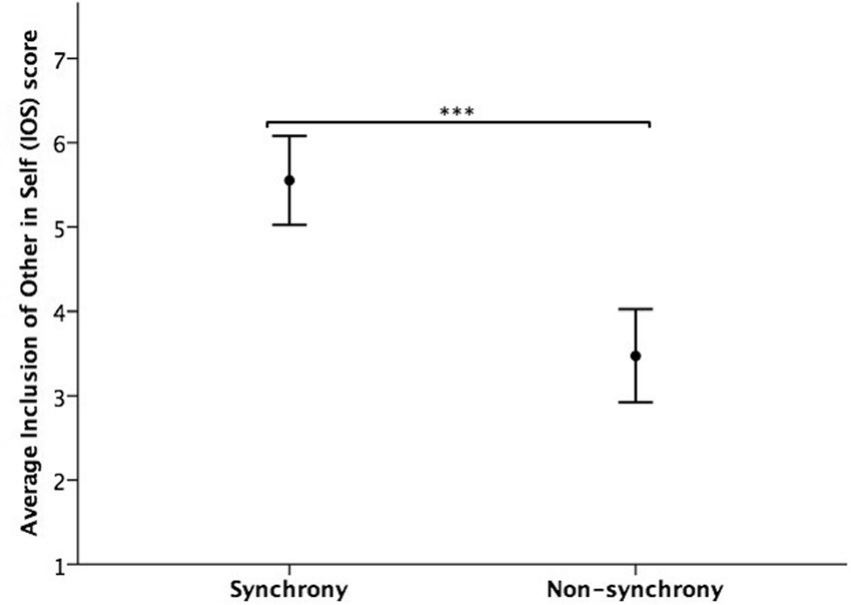
Mean (95% CI) IOS score (1–7 likert scale) in each movement condition.

**Figure 2:**
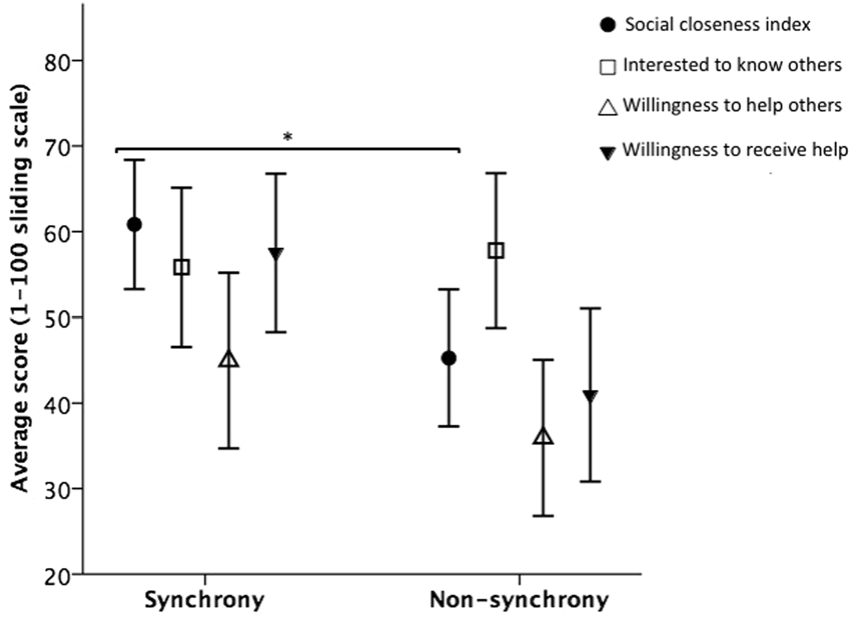
Mean scores (95% CI) for social closeness index, interest in knowing the others, and willingness to offer and receive help from others, *n* = 76, *p < 0.05.

Mean ratings of inclusion of other in self (IOS, 1 – 7 scale) were higher in the synchrony condition (*M* = 5.55, SD = 1.61) than the non-synchrony condition (*M* = 3.47, SD = 1.67; ANOVA (2-tailed): *F*(1, 72) = 16.62, *p* < 0.001, η_p_^2^ = 0.188, Fig. 1). Analyses also revealed a significant difference between movement conditions in the social closeness index scores, with the synchrony condition (*M* = 60.83, SD = 22.98) scoring higher than the non-synchrony condition (*M* = 45.26, SD = 24.3; ANOVA (2-tailed): *F*(1, 72) = 4.27, *p* = 0.043, η_p_^2^ = 0.056, Fig. 2). Analyses did not reject the null hypothesis of no significant difference between conditions in how interested participants were to know the others from their movement task, and in their hypothetical willingness to offer or accept help to/from others if needed (Supplementary Information 4, Tables S1 and S8).

### Mimicry during idle period

There was no significant difference between conditions in incidents of mimicry following the VR activity. Eleven participants in each condition were coded as engaging in mimicry.

### Proximity

A Mann-Whitney U test (2-tailed) on proximity change (mean of post- minus pre- positions for each stage) to each of the other virtual characters yielded no significant difference between conditions for either one character (*M*_*Sync*_ = −0.002 m, SD = 0.025, *M*_*N-Sync*_ = −0.001 m, SD = 0.014, *n* = 76, *p* = 0.801) or the other (*M*_*Sync*_ = 0.001 m, SD = 0.032, *M*_*N-Sync*_ = 0.002 m, SD = 0.019, *n* = 76, *p* = 0.811). Similarly, overall (averaging proximities across the task stages), there was no significant difference between participants’ average distance from one character (*M*_*Sync*_ = 1.395 m, SD = 0.080, *M*_*N-Sync*_ = 1.415 m, SD = 0.084, *n* = 76, *p* = 0.228) or the other (*M*_*Sync*_ = 1.404 m, SD = 0.071, *M*_*N-Sync*_ = 0.391 m, SD = 0.081, *n* = 76, *p* = 0.280) in the two conditions (Supplementary Information 4, Tables S9 for full results).

## Discussion

This study used first-person immersive VR technology to test the effects of synchrony on social closeness. Participants in the synchrony condition reported greater social closeness compared to participants in the non-synchrony condition. These results provide robust evidence of the relative effects of movement synchrony and non-synchrony. Importantly, instructions to participants were identical between conditions. By covertly coding the timing of the other virtual human characters’ movements in relation to participants’ movements, we manipulated synchrony without relying on explicit instruction that participants actively move in or out of time with their co-participants. Further, the VR set-up eliminated any requirement to use individual stimuli, such as personal headphones, that potentially increase a sense of separation.

Notably, the positive effect of synchrony on social closeness was evident even though participants who synchronized reported the task as more difficult than those who were in the non-synchrony condition. Participants took part in a ‘together’ movement activity in which synchrony or non-synchrony emerged in the absence of any express joint intentions or goals to match or un-match movements with the others. In the context of this ‘together’ group activity, it is unlikely that participants perceived non-synchrony as antagonistic or incongruent in relation to their own movement. Movements were silent, slow and controlled and did not resemble any real-world activity in which interpersonal synchrony or temporal coordination is a typical expectation (as, for example, in drumming and rowing). The results support the hypothesis that synchronous movement boosts feelings of social closeness. It is still possible, however, that non-synchrony and synchrony both contribute to effects, with synchrony enhancing feelings of closeness and non-synchrony diminishing them. Future studies should incorporate baseline measures to isolate positive effects of synchrony and/or negative effects of non-synchrony. Furthermore, it is not clear from our study whether there is a ‘threshold’ degree of synchrony that would be required to trigger the positive social effects associated with synchrony in this and other studies. In our design, we delineated synchrony from non-synchrony on the basis of varying temporal delays in movement execution, with tight coupling in the synchrony condition (less than 0.58 second delay) and a greater delay in the non-synchrony condition (1.67–4.33 seconds). Future studies could use circular statistics and measure of mean vector length scores^27^ to determine at what degree of delay the synchrony-related effects are reduced.

Contrary to our predictions, analyses of behavioral measures of spatial proximity and mimicry revealed no significant differences between conditions. Participants in the synchrony condition did not move closer to the other two characters over the course of the activity, nor mimic them more after the activity. The lack of any observable differences in these behavioral measures may be due to the novelty of the environment, which could be improved with more use. The full body suit and headgear possibly constrains all movement at first, especially movement extraneous to the task. Spontaneous behavior might increase with greater opportunity to become accustomed to the equipment.

Most participants did not think that the other characters were controlled by real people, which may have influenced responses to questions concerning behavior (e.g., interest in meeting in the future, likelihood of assisting with money). Greater social closeness in the synchrony condition suggests that emotional bonding effects still occur in the absence of an explicit belief that one is moving in the presence of other people. The reported feelings of social closeness may derive from implicit processes involved in social perception and ‘self-other’ overlap. Other research has demonstrated strong socio-cognitive and behavioral effects in immersive VR environments, even when virtual bodies are evidently ‘fictional’ in some sense. Peck *et al*.^28^ found that virtual embodiment of light-skinned participants in dark-skinned characters led to a reduction in implicit racial biases. A study by Freeman *et al*.^29^ found that reduction of a person’s height in a VR environment leads to negative social comparison and paranoia. Furthermore, VR technologies have been successfully used to investigate effects of social responsiveness on attachment and paranoia and to facilitate positive social experiences for those suffering from paranoia and persecutory delusions^30,31^.

Although the present study did not compare the positive effects of synchrony in virtual and real-world environments, or between healthy and clinical samples, the findings are potentially relevant for the development of therapies for people who have difficulty developing social closeness with others, or disorders that interfere with movement and embodied interaction. Our results suggest that VR experiences could be an effective tool in social-cognitive therapies in which patients benefit from positive social interactions in a virtual space. In real-world interactions, moving in synchrony with others appears to establish social connection among adults^18,20^, teenagers^19^ and children^32^, even across existing group divides^25^. Compared to moving alone or non-synchronously with others, synchronous movement is also associated with elevated pain thresholds^18,19,33,34^. The positive social and physiological effects of such ‘mutual motion’ suggest that activities involving synchronous group movement may alleviate some of the negative effects of social isolation, including attenuation of threat and stress^35^.

Further research and the much-anticipated rolling out of VR technologies in everyday, online, and virtual social interactions will help to reveal the scope of possibilities for virtual social networks, support, and interaction to improve socio-cognitive abilities and health. Forbes *et al*.^36^ reported that VR characters can induce mimicry in neurotypical and autistic participants, though the latter responded to a lesser extent. Ramachandran & Seckel^37^ have suggested that perception of synchronized movement using mirrors could stimulate the mirror neuron system and the co-activation action perception networks. A VR set-up similar to that of the current study could offer a richer interpersonal experience with other individuals, potentially facilitating social connection and empathy. Neufield and Chakrabarti^38^ showed a stronger link between mimicry and social reward among participants who had higher trait empathy. In combination with other interventions, VR interactions could potentially strengthen links between mimicry or synchrony and social reward, and help confirm whether higher empathy is a cause or consequence of the mimicry-reward link.

VR environments provide a valuable platform for investigating the socio-cognitive effects of human behavior and interaction. Our findings suggest that the effects of movement synchrony are robust in a VR environment. Synchrony is methodologically simple to establish and control using VR, involving no or minimal time delay in the mirroring of movements performed by the participant. As technologies become more readily available and pricing more competitive, synchronous and coordinated activities in an immersive VR environment could be an effective laboratory and therapy tool for measuring and fostering feelings of social closeness.

## Methods

### General study design

Participants were informed that the experiment aimed to investigate the effect of lighting in a VR social setting, and people’s responses to VR experiences. Participants attended alone and were told that they would be joined by others in the VR task. The other virtual characters did not represent real people and this was not explicitly confirmed to the participants. Participants were guided through the study by a hypothesis-blind experimenter, who was trained in using the VR technology. After obtaining informed consent, participants completed a computer-based questionnaire (Supplementary Information 5), including demographic questions, a mini-International Personality Item Pool (IPIP) scale^39^, and a Positive and Negative Affect Scale^40^. In a private cubicle, they learned three basic upper body movements from a video (Supplementary Information 6). Movements were simple arm reaches performed in a repetitive and slow manner. Each movement was named and participants rated their recall confidence for label-movement pairings at the end of the training video.

Participants entered the VR lab and were fitted with a head-mounted display (HMD). They were familiarized with their virtual body seen from first person perspective. Shortly afterward, two virtual human characters (avatars) appeared in the VR, and a pre-recorded audio track informed the participants: “*The group movement task is about to start. You are now together with the others and you will join the group to do the movements you learnt earlier. Please turn so that you can see the other avatars. The audio instructions will tell you what movement to do, and when to change to the next movement in the sequence”* (Supplementary Information 5). The three virtual characters stood in a triangle facing inwards and the movement task lasted three minutes. This consisted of three repetitions of the movement sequence (‘movement stages’), during which each movement was performed for 20 seconds before moving on to the next movement at the instruction of the audio track. After the movement sequence, the audio track instructed participants to wait for the researcher to come to remove their HMD. During this time (‘idle stage’), the other two characters each assumed a pre-coded ‘idle’ pose (see *‘Mimicry during idle phase’* below), one lifting their hand to their chin (Supplementary Information 7), and the other standing with their hand on their hip (Supplementary Information 8).

Participants returned to the computer and completed a post-activity questionnaire (Supplementary Information 5), which included a series of questions measuring social closeness, Inclusion of Other in Self (IOS), and cooperation (see *‘Self-report social closeness’* below), the PANAS, and questions regarding participants’ experience of the experiment. Participants also rated their success in following the audio instructions, how synchronized their movements had been with the other characters, how often it felt like they were following the movements of the other characters or were being followed by the other characters. Although it was not explicitly stated that the other characters were controlled by real people, it was implied that this was a social activity, and participants were asked to rate the extent to which they *felt* the other characters were controlled by real people and whether it *felt* like they were with other people during the VR movement task. Participants then received their compensation and were debriefed.

### VR environment

**Figure 3:**
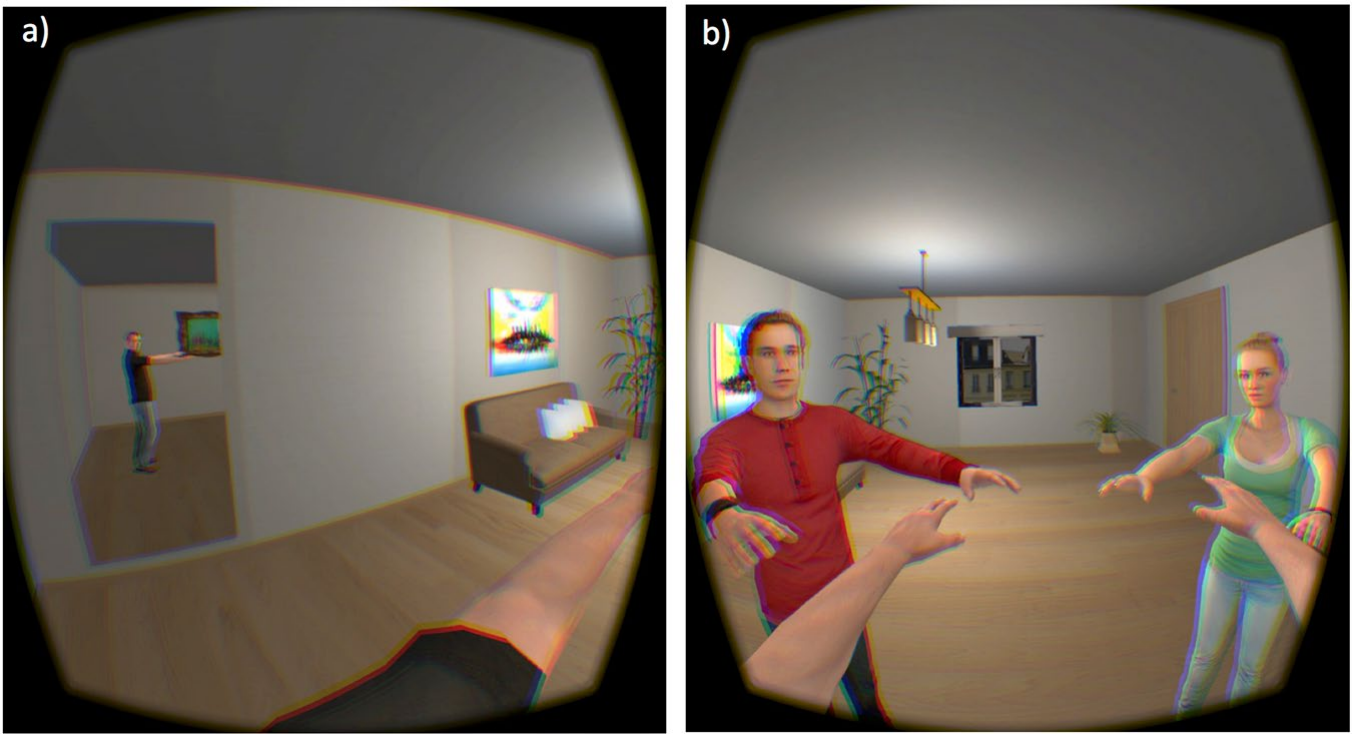
A screen-capture image from a video of first-person perspective in the VR environment (**a**) in the embodiment stage (seeing their virtual body reflected in a mirror) and (**b**) with the other two virtual characters during the movement task. This image appeared in focus when viewed through the VR headset.

In the VR lab (width: 2.96 m, length: 3.4 m - back wall to curtain - height: 2.87 m), participants were fitted with an Optitrack full body motion capture suit (37 markers) and an Oculus Rift DK2 (https://www3.oculus.com/en-us/dk2/) HMD. This has a nominal field-of-view of 100°, with a resolution of 960 × 1080 per eye displayed at 60 Hz. The virtual environment (a simple room with basic decoration, see Fig. 3) was implemented on the Unity3D platform (http://unity3d.com/unity). Participants’ virtual body skeletons were rendered using the Motive Software (https://www.naturalpoint.com/optitrack/products/motive/body/features-specs.html) via infrared technology which was implemented with a 12-camera truss setup by OptiTrack. One male and one female virtual human character was selected from an existing bank developed at EVENT Lab and used, respectively, for all male and female participants. An additional male and female virtual human character were used for the two co-participant characters. Approximate character age was selected to fall within the normal range of the student population from which participants were recruited, and their look was casual. At the start of the VR experience, participants were asked to turn to their left, where a mirror (reflecting back the image of their virtual body) aided their sense of body ownership (Fig. 3a).

### Manipulation of synchrony

Participants were randomly assigned to either the synchrony (*n* = 38) or non-synchrony (*n* = 38) condition. Participants received identical instructions in both conditions. During the VR task, the movement of each of the other two characters was determined by cloning the upper-body of the participant’s virtual body and applying a pre-coded delay in timing (Supplementary Information 9). In the synchrony condition, the other characters’ movements were delayed by a small amount (15–35 frames/second, i.e. 0.25–0.58 second delay) to induce a more ‘natural’ feel (as perfect cloning would be obvious to the participant and may bias the participants’ experience of the characters; von Zimmermann, *personal communication*). In the non-synchrony condition, the other characters’ movements were delayed by a greater amount (100–260 frames/second, i.e. 1.67–4.33 second delay). The operationalization of non-synchrony to constitute a delay in movement is in accordance with previous research on the effects of synchrony^20,27^. To avoid a situation where the other two characters were in synchrony with each other (independent of the participant), which may have caused the participant to view the other two characters as a being part of their own separate group, the other characters were coded to have different delays from each other in both conditions. The degree of delay varied within the above-mentioned ranges and for each character throughout the movement task, with changes in the delays initiated at the start of each stage of the movement task (i.e. when the participant changed from one movement to the next in the sequence).

### Dependent measures

#### Self-report social closeness

Social closeness was measured using self-report scales (Supplementary Information 5). These included an adapted version of the IOS scale^41^, and, on a sliding scale of 1–100, questions on connectedness^5^, likeability^1^, and ratings of similarity in personality^3^. These three items were subjected to a principal axis factoring to assess the dimensionality of the data (KMO = 0.687, *p* < 0.001). One factor was extracted (eigenvalue = 2.025, 67.50% of variance, see Supplementary Information 4, Table S4 for correlation matrix). The connectedness, likeability, and similarity in personality scores were averaged to create a ‘social closeness index’ (Cronbach’s *α* = 0.76). Participants were also asked to rate their interest in meeting the other participants from their session, their likelihood of helping one of the others in their session by lending them 10 Euro if they needed it, and the participant’s likelihood of accepting help from one of the others by borrowing 10 Euro if it were offered (each on a 1–100 sliding scale).

#### Proximity

The VR software stored each participant’s physical proximity (in meters) to both virtual characters at each frame of the VR session. To determine changes in the participant’s proximity to the two characters, the average distance to each was calculated for each stage of the VR session (‘Meeting stage’ and ‘Movement stages 1–9’). As there was some small variation in the starting position of participants, the average distance to character 1 and character 2 in the first stage (‘Meeting stage’) was taken as a ‘start distance’ as this occurred before the movement task began.

#### Mimicry during idle period

At the end of the movement task the other characters assumed idle positions (Supplementary Information 7 or 8). Two independent coders rated instances of mimicry (Supplementary Information 1) and ratings were used to assess whether there were more instances of mimicry following synchrony than non-synchrony.

### Statistical analysis

Data were analyzed using IBM SPSS (Version 23) and were checked for normality using Kolmogorov-Smirnov tests. Ratings of willingness to help others, receive help and the combined social closeness index data were normally distributed in both movement conditions (*p* > 0.05; Supplementary Information 4, Table S2). The change in positive and negative affect (calculated as a post-score minus pre-score) were non-normally distributed (Supplementary Information 4, Table S2). Almost all variables had homogenous variance (with the exception of self-assessment of synchrony with the other characters, and ratings of how often participants felt they were following the other two and how often they felt they were being followed; Supplementary Information 5, Table S2). Log transformations did not normalize the data. Parametric (univariate ANOVA) analyses were used when the data conformed to the assumptions of this test; otherwise, the data were analyzed using Mann-Whitney U Test or Chi-squared Test where appropriate. Due to the importance of certain covariates (see results), non-normally distributed dependent variables were also checked with parametric tests (as non-parametric analyses did not allow for the inclusion of these covariates). The residuals of the proximity data were non-normally distributed for both characters (Supplementary Information 4, Table S3), and non-parametric analyses were required. The average distance between the participant and each character at each stage was corrected for starting position by subtracting the start distance from the average distance to each character in each stage.

### Research Disclosure Statement

The authors confirm that the total number of excluded observations and the reasons for making these exclusions have been reported in the Method section, and that all independent variables or manipulations, whether successful or failed, have been reported in the Method section. Furthermore, the authors confirm that all dependent variables or measures that were analyzed for this article’s target research question have been reported in the Methods section. All data analysed during this study are included in this published article (and its Supplementary Information files).

### Ethical Considerations and conflicts of interest

Ethics approval was obtained from the University of Oxford’s CUREC and the Bioethical Committee of the University of Barcelona. Participants signed an informed consent document prior to taking part in the study.

## Electronic supplementary material

Supplementary Info File 1

Supplementary Information 2

Supplementary Information 3

Supplementary Information 6

Supplementary Information 7

Supplementary Information 8

Dataset 1
