# Supplementary material for: Synchrony and social connection in immersive Virtual Reality
Source: Sci Rep. 2018 Feb 27;8:3693. doi: 10.1038/s41598-018-21765-4 (PMC5829252; doi:10.1038/s41598-018-21765-4)
Supplement: Supplementary file 1 — Supplementary Info File 1 [file 41598_2018_21765_MOESM1_ESM.docx]

**Supplementary Information 1**

## 1.1 Coding scheme

**PART 1. Coding of Non-/Synchronous Movements During a Movement Activity**

**Aim of the analysis**

To determine whether three avatars are synchronized or unsynchronized in their movements.

**What to analyse**

A total of 76 videos amounting to app. 170 minutes.

In each video, three virtual humans (avatars) are displayed standing side by side (see Fig.S1). This is a playback of an actual movement task in which people wore motion capture suits and performed a simple movement sequence with others in a virtual reality (VR) environment in two conditions: synchronous movement and non-synchronous movement. In the VR environment, people were represented by avatars. Each participant’s virtual character had a ‘first-person’ perspective, and the group stood in a triangle facing inwards.

In the video you will watch, a program has placed the virtual characters side by side so that you can better assess whether they are, in your opinion, in the synchronous or non-synchronous condition. Once you have loaded the file, click ‘Start’ and drag the round button to the time when they start the movement task (approx. just beneath the end of the ‘Load File button’). Watch the video until their arms return to hang naturally by their sides (beneath the ‘Pause’ button, approx. 1 cm before the end).


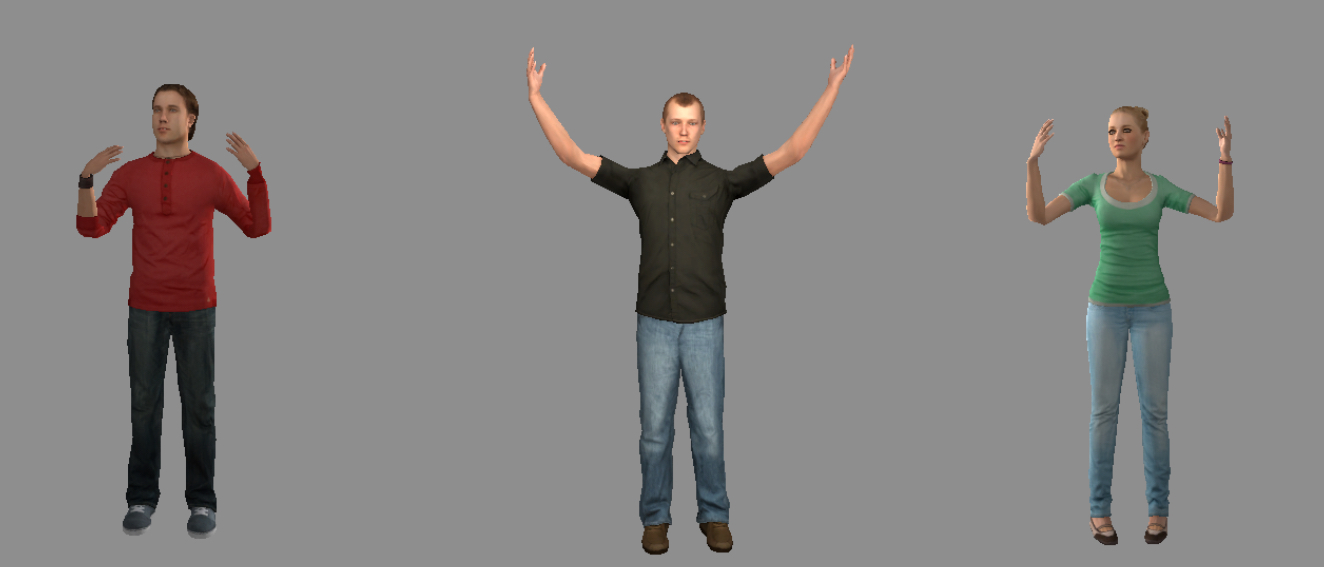


Figure S1. A screen-capture image of the playback software used by the independent coders. The central virtual character represents the participant, in this case in the non-synchrony condition.

**How to do the analysis**

*Primary Coding:*

For each video (with file name *‘PlayBack.txt’*), you will guess which condition (sync/non-sync) the group was allocated. Under the column ‘*condition_check’*, you will code whether the virtual characters’ movements were temporally synchronized, i.e. the characters are doing the same movements at the same time (code ‘1’), or not synchronized, i.e. the characters are not doing the same movements at the same time (code ‘2’).

*Non-synchrony subsidiary coding:*

Within those groups that you identify as non-synchronous, it may be that synchrony arises between two or more of the virtual characters for a period of the task. In this case, please record your estimate how much of the time this synchrony occurred (less than half the time = code ‘L’; about half the time = code ‘H’; more than half the time = code ‘M’).

| **Primary Code** | | **Description** | |
| --- | --- | --- | --- |
| 1 | | The avatars movements are synchronized | |
| 2 | | The avatars movements are not in synchrony | |
|  | **Non-Synchrony subsidiary coding:** | |  |
|  | L | Less than half the time | |
|  | H | About half the time | |
|  | M | More than half the time | |

**PART 2. Coding of Similar Movements during the idle phase**

**Aim of the analysis**

To code the similarity of movement of the participant to the other virtual characters during an idle waiting stage after the movement activity.

**What to analyse**

A total of 76 videos amounting to app. 40 minutes.

At the end of the movement task, the virtual characters are awaiting further instruction, and are free to do any movement with their bodies. As before, the video you will watch will show the three virtual characters side by side. You will assess the idling movement of the participant (the central character on your screen) relative to the two other characters (on either side of the central character). The two other characters do the following movements:

Male character: Bends his arm at the elbow and raises his hand to his face.

Female character: Bends her arm at the elbow and places her hand on her hip.

**How to do the analysis**

You will watch the 30 second idle period (these data files are named *‘Waiting.txt’* at the end) and under the column ‘*idle_check’* will code the central virtual character (participant)’s movements according to the following scale:

| **Code Description** | |
| --- | --- |
| 1 | The central avatar (participant) raises their hand to their face, or towards their face. [Note: If the participant does not complete this motion fully, but raises their hand toward their face and higher than their bent elbow, code the movement as ‘1’.] |
| 2 | The central avatar (participant) bends at least one of their arms and places their hand on their hip/s, or moves it towards their hip/s or midriff area. |
| 3 | The central avatar (participant) does both ‘1’ and ‘2’ above. |
| 0 | The central avatar (participant) does not do ‘1’, ‘2’ or ‘3’ above. |

## Details of manipulation check results

According to the subsidary coding as to whether one or more of the virtual characters were synchronized for less than half the time, half the time, and more than half the time, for 7/38 non-synchrony-asigned participants, one or both of the coders assessed that synchrony occurred for half or more than half of the time. These 7 instances were then checked according to how the participants had themselves rated their own degree of synchrony (where 1 = *never* and 100 = *always*). Two participants who had been assigned to the synchrony condition scored their degree of synchrony with other avatars as ≤50/100 and 12 in the non-synchrony condition rated their degree of synchrony as >50/100. Neither instance had been simialrly coded by the independent raters. As there are no established thresholds for how much synchrony (or non-synchrony) overall is required for an activity to be perceived as synchronous, and the coders’ condition-wise ratings (Part 1 above) in all of these cases agreed with the pre-established condition, we retained conditions as originally assigned for analysis.

## Supplementary Information 2

## Video Sample of Synchrony <SI2_VR sample of Synchrony.mp4>

## Supplementary Information 3

## Video Sample of Non-Synchrony <SI3_VR sample of NonSynchrony.mp4>

## Supplementary Information 4

## Additional Statistics

##### Descriptive statistics

Table S1. Mean, standard deviation, median and standard error for all variables, split according to movement condition. Items marked * were measured on a 1-7 scale; † were measured on a 1-5 scale; and all other items on 1-100 scale.

| **Dependent variable** | **Movement condition** | **Mean** | **Standard deviation** | **Median** | **Standard Error** |
| --- | --- | --- | --- | --- | --- |
| *** IOS** | Synchrony | 5.550 | 1.606 | 6.000 | .260 |
|  | Non-synchrony | 3.470 | 1.672 | 3.000 | .271 |
| **Combined social closeness** | Synchrony | 60.833 | 22.984 | 63.000 | 3.729 |
|  | Non-synchrony | 45.263 | 24.297 | 43.000 | 3.941 |
| **Connectedness** | Synchrony | 72.680 | 24.429 | 79.000 | 3.963 |
|  | Non-synchrony | 45.76 | 26.660 | 47.000 | 4.325 |
| **Likeability** | Synchrony | 64.820 | 29.629 | 72.500 | 4.806 |
|  | Non-synchrony | 57.370 | 28.290 | 60.50 | 4.589 |
| **Similarity in personality** | Synchrony | 45.000 | 34.079 | 45.500 | 5.528 |
|  | Non-synchrony | 32.660 | 30.110 | 21.500 | 4.884 |
| **Interested to know the others** | Synchrony | 55.840 | 28.263 | 55.000 | 4.585 |
|  | Non-synchrony | 57.790 | 27.474 | 65.500 | 4.457 |
| **Willingness to help others** | Synchrony | 44.950 | 31.152 | 46.500 | 5.053 |
|  | Non-synchrony | 35.920 | 27.765 | 29.500 | 4.504 |
| **Willingness to receive help** | Synchrony | 57.530 | 28.126 | 56.500 | 4.563 |
|  | Non-synchrony | 40.92 | 30.721 | 37.000 | 4.984 |
| **Success at following instructions** | Synchrony | 79.170 | 20.033 | 85.000 | 3.386 |
|  | Non-synchrony | 78.510 | 16.172 | 80.000 | 2.659 |
| **Degree of synchrony with others** | Synchrony | 89.400 | 19.036 | 96.000 | 3.218 |
|  | Non-synchrony | 37.810 | 27.015 | 32.000 | 4.441 |
| **Being followed by avatars** | Synchrony | 88.830 | 19.940 | 98.000 | 3.371 |
|  | Non-synchrony | 46.780 | 33.590 | 55.00 | 5.522 |
| **Following avatars** | Synchrony | 29.910 | 34.702 | 12.000 | 5.866 |
|  | Non-synchrony | 25.430 | 25.464 | 14.000 | 4.186 |
| **Other avatars controlled by real people** | Synchrony | 31.090 | 32.010 | 18.000 | 5.411 |
|  | Non-synchrony | 32.300 | 30.750 | 19.000 | 5.055 |
| **In the presence of other people** | Synchrony | 44.340 | 32.019 | 49.00 | 5.412 |
|  | Non-synchrony | 39.540 | 31.168 | 41.000 | 5.124 |
| **Confidence (remembering movements)** | Synchrony | 88.050 | 11.761 | 88.500 | 1.908 |
|  | Non-synchrony | 86.630 | 12.918 | 89.000 | 2.096 |
| †**Extraversion** | Synchrony | 13.890 | 1.997 | 14.000 | .328 |
|  | Non-synchrony | 14.130 | 1.961 | 14.000 | .318 |
| †**Conscientiousness** | Synchrony | 12.060 | 1.913 | 13.000 | .314 |
|  | Non-synchrony | 12.160 | 1.424 | 12.000 | .231 |
| †**Neuroticism** | Synchrony | 11.810 | 1.793 | 12.000 | .295 |
|  | Non-synchrony | 11.260 | 1.750 | 11.000 | .284 |
| †**Intellect** | Synchrony | 10.050 | 1.649 | 10.000 | .271 |
|  | Non-synchrony | 10.370 | 1.567 | 10.000 | .254 |
| †**Agreeableness** | Synchrony | 11.460 | 1.502 | 12.000 | .247 |
|  | Non-synchrony | 11.110 | 1.429 | 11.000 | .232 |
| **Fun** | Synchrony | 77.470 | 21.121 | 83.500 | 3.426 |
|  | Non-synchrony | 70.210 | 22.954 | 70.500 | 3.724 |
| **Awkward** | Synchrony | 11.610 | 20.472 | 4.000 | 3.321 |
|  | Non-synchrony | 11.370 | 16.744 | 4.000 | 2.716 |
| **Difficulty** | Synchrony | 8.450 | 11.767 | 4.000 | 1.909 |
|  | Non-synchrony | 4.160 | 8.079 | 1.000 | 1.311 |
| **Enjoyment** | Synchrony | 75.680 | 20.551 | 76.000 | 3.334 |
|  | Non-synchrony | 77.530 | 15.782 | 79.000 | 2.560 |
| †**Positive Affect (start)** | Synchrony | 3.140 | .533 | 3.200 | .090 |
|  | Non-synchrony | 3.150 | .584 | 3.200 | .096 |
| †**Negative Affect (start)** | Synchrony | 1.350 | .388 | 1.200 | .066 |
|  | Non-synchrony | 1.380 | .357 | 1.400 | .059 |
| †**Positive Affect (end)** | Synchrony | 3.610 | .633 | 3.800 | .107 |
|  | Non-synchrony | 3.520 | .654 | 3.600 | .108 |
| †**Negative Affect (end)** | Synchrony | 1.190 | .310 | 1.000 | .052 |
|  | Non-synchrony | 1.210 | .290 | 1.000 | .048 |
| †**Positive Affect (change)** | Synchrony | .460 | .596 | .400 | .101 |
|  | Non-synchrony | .370 | .513 | .600 | .084 |
| †**Negative Affect change)** | Synchrony | -.170 | .396 | .000 | .067 |
|  | Non-synchrony | -.170 | .310 | -.200 | .051 |

##### Normality testing and homogeneity of variance

Kolmogorov-Smirnov and Levenes’ tests indicated that much of the data were normally distributed with homogenous variance (see Table S2 and S3). Log transformations did not result in normality for any of the variables that were not normal.

Table S2. P-values for Kolmogorov-Smirnov normality test and Levene's test of homogenous variance on all variables (*n_syn_*=38, *n_non-syn_* = 38).

| **Dependent variable** | **Movement condition** | **Start score**  **(p-value)** | **End score**  **(p-value)** | **Change**  **(p-value)** | **Homogeneity of variance**  **(p-value)** |
| --- | --- | --- | --- | --- | --- |
| **IOS** | Synchrony | N/A | .000 | N/A | .770 |
|  | Non-synchrony | N/A | .001 | N/A |  |
| **Connectedness** | Synchrony | N/A | .094 | N/A | .471 |
|  | Non-synchrony | N/A | .200 | N/A |  |
| **Likeability** | Synchrony | N/A | .000 | N/A | .583 |
|  | Non-synchrony | N/A | .200 | N/A |  |
| **Similarity in personality** | Synchrony | N/A | .200 | N/A | .345 |
|  | Non-synchrony | N/A | .011 | N/A |  |
| **Interested to know the others** | Synchrony | N/A | .200 | N/A | .843 |
|  | Non-synchrony | N/A | .010 | N/A |  |
| **Combined social closeness** | Synchrony | N/A | .200 | N/A | .665 |
|  | Non-synchrony | N/A | .200 | N/A |  |
| **Willingness to help others** | Synchrony | N/A | .070 | N/A | .451 |
|  | Non-synchrony | N/A | .069 | N/A |  |
| **Willingness to receive help** | Synchrony | N/A | .200 | N/A | .777 |
|  | Non-synchrony | N/A | .200 | N/A |  |
| **Success at following instructions** | Synchrony | N/A | .002 | N/A | .663 |
|  | Non-synchrony | N/A | .141 | N/A |  |
| **Degree of synchrony with others** | Synchrony | N/A | .000 | N/A | .001 |
|  | Non-synchrony | N/A | .200 | N/A |  |
| **Being followed by avatars** | Synchrony | N/A | .000 | N/A | .000 |
|  | Non-synchrony | N/A | .022 | N/A |  |
| **Following avatars** | Synchrony | N/A | .000 | N/A | .029 |
|  | Non-synchrony | N/A | .000 | N/A |  |
| **Other avatars controlled by real people** | Synchrony | N/A | .002 | N/A | .796 |
|  | Non-synchrony | N/A | .002 | N/A |  |
| **In the presence of other people** | Synchrony | N/A | .141 | N/A | .886 |
|  | Non-synchrony | N/A | .047 | N/A |  |
| **Confidence (remembering movements)** | Synchrony | .022 | N/A | N/A | .679 |
|  | Non-synchrony | .030 | N/A | N/A |  |
| **Extraversion** | Synchrony | .008 | N/A | N/A | .919 |
|  | Non-synchrony | .002 | N/A | N/A |  |
| **Conscientiousness** | Synchrony | .118 | N/A | N/A | .132 |
|  | Non-synchrony | .320 | N/A | N/A |  |
| **Neuroticism** | Synchrony | .017 | N/A | N/A | .915 |
|  | Non-synchrony | .063 | N/A | N/A |  |
| **Intellect** | Synchrony | .051 | N/A | N/A | .781 |
|  | Non-synchrony | .041 | N/A | N/A |  |
| **Agreeableness** | Synchrony | .001 | N/A | N/A | .839 |
|  | Non-synchrony | .005 | N/A | N/A |  |
| **Fun** | Synchrony | N/A | .048 | N/A | .609 |
|  | Non-synchrony | N/A | .015 | N/A |  |
| **Awkward** | Synchrony | N/A | .000 | N/A | .846 |
|  | Non-synchrony | N/A | .000 | N/A |  |
| **Difficulty** | Synchrony | N/A | .000 | N/A | .068 |
|  | Non-synchrony | N/A | .000 | N/A |  |
| **Enjoyment** | Synchrony | N/A | .198 | N/A | .371 |
|  | Non-synchrony | N/A | .200 | N/A |  |
| **Positive Affect** | Synchrony | .200 | .019 | .040 | .903 |
|  | Non-synchrony | .135 | .200 | .002 |  |
| **Negative Affect** | Synchrony | .001 | .000 | .001 | .170 |
|  | Non-synchrony | .000 | .000 | .001 |  |

Table S3. P-values for Kolmogorov-Smirnov normality test and Levene's test of homogenous variance on proximity data (*n_syn_*=38, *n_non-syn_* = 38)

|  | **Movement condition** | **Distance before the movement task**  **(p-value)** | **Distance at end of movement task**  **(p-value)** | **Average Distance during movement task (p-value)** | **Change in distance**  **(p-value)** | **Standardized residual of end distance**  **(p-value)** | **Homogeneity of variance of residuals**  **(p-value)** |
| --- | --- | --- | --- | --- | --- | --- | --- |
| **Virtual character 1** | Synchrony | .009 | .000 | .016 | .000 | .000 | .227 |
|  | Non-synchrony | .001 | .001 | .010 | .003 | .140 |  |
| **Virtual character 2** | Synchrony | .000 | .094 | .011 | .000 | .000 | .235 |
|  | Non-synchrony | .001 | .200 | .000 | .002 | .002 |  |

##### Additional statistical results

Table S4. Correlation matrix as part of Exploratory Factor Analysis (principle axis factoring).

|  |  | Connectedness | Likability | Similar in personality |
| --- | --- | --- | --- | --- |
| **Correlation** | Connectedness | 1.000 | .470 | .495 |
|  | Likeability | .470 | 1.000 | .571 |
|  | Similar in personality | .495 | .571 | 1.000 |
|  | Connectedness |  | .000 | .000 |
| **Sig. (1-tailed) p** | Likeability | .000 |  | .000 |
|  | Similar in personality | .000 | .000 |  |

Determinant = .474

Table S5. Baseline comparisons between movement conditions for ANOVA analyses and non-parametric Mann-Whitney U tests where appropriate.

|  | Sum of Squares | Df | Mean Square | *F* | *p* | *p (non-parametric)* |
| --- | --- | --- | --- | --- | --- | --- |
| **Extraversion** | N/A | 1 | N/A | N/A | N/A | .536 |
| **Agreeableness** | N/A | 1 | N/A | N/A | N/A | .137 |
| **Contentiousness** | 5.564 | 1 | 5.564 | 1.964 | 0.165 | N/A |
| **Neuroticism** | N/A | 1 | N/A | N/A | N/A | .225 |
| **Intellect** | N/A | 1 | N/A | N/A | N/A | .337 |
| **Confidence in ability to perform movements** | N/A | 1 | N/A | N/A | N/A | .730 |

Table S6. The effect of movement condition on various measures of participants’ experience of the experiment: ANOVA analyses and, as appropriate, non-parametric Mann-Whitney U tests.

|  | Sum of Squares | Df | Mean Square | *F* | *p* | *p (non-parametric)* |
| --- | --- | --- | --- | --- | --- | --- |
| **Fun** | N/A | 1 | N/A | N/A | N/A | .152 |
| **Difficulty** | N/A | 1 | N/A | N/A | N/A | .001 |
| **Awkwardness** | N/A | 1 | N/A | N/A | N/A | .933 |
| **Enjoyment** | 64.47 | 1 | 64.47 | .192 | .662 | N/A |
| **Success at following instructions** | N/A | 1 | N/A | N/A | N/A | .629 |
| **Avatars controlled by real people** | N/A | 1 | N/A | N/A | N/A | .930 |
| **With others in the VR** | N/A | 1 | N/A | N/A | N/A | .589 |
| **Following other avatars** | N/A | 1 | N/A | N/A | N/A | .954 |
| **Being followed by other avatars** | N/A | 1 | N/A | N/A | N/A | .000 |
| **Synchronized with other avatars** | N/A | 1 | N/A | N/A | N/A | .000 |

Table S7. Results for repeated measures ANOVA and non-parametric Mann-Whitney U test of change in positive and negative affect.

|  |  | | Sum of Squares | Df | Mean Square | *F* | *p* | *Partial Eta Squared (*η_p_^2^) | *p (non-parametric)* |
| --- | --- | --- | --- | --- | --- | --- | --- | --- | --- |
| **Time point** | | Positive affect | 6.224 | 1 | 6.224 | 40.000 | <.001 | .367 | N/A |
|  |  | Negative affect | .907 | 1 | .907 | 4.462 | <.001 | .178 | N/A |
| **Time point *Condition** | | Positive affect | .094 | 1 | .094 | .604 | .440 | .009 | .729 |
|  |  | Negative affect | .006 | 1 | .006 | .098 | .755 | .001 | .411 |

Table S8. The effect of movement condition on various prosociality measures (*n* = 76). ANOVA analyses and non-parametric Mann-Whitney U tests where appropriate (including covariates in parametric analyses).

|  | Sum of Squares | Df | Mean Square | *F* | *p* | *Partial Eta Squared (*η_p_^2^) | *p (non-parametric)* |
| --- | --- | --- | --- | --- | --- | --- | --- |
| **IOS** | 42.082 | 1 | 42.082 | 16.617 | .000 | .188 | .022 |
| **Social closeness index** | 2205.468 | 1 | 2205.468 | 4.265 | .043 | .056 | N/A |
| **Interested to know the others** | 250.222 | 1 | 250.222 | .319 | .574 | .004 | .585 |
| **Willingness to help the others** | 1541.505 | 1 | 1541.505 | 1.737 | .192 | .024 | N/A |
| **Willingness to receive help** | 2323.468 | 1 | 2323.468 | 2.615 | .110 | .035 | N/A |

Note that although the willingness to receive help measure was significantly higher for those in the synchrony condition (*M* = 57.53, SD = 28.13) compared to non-synchrony condition (*M* = 40.92, SD = 30.72) when the covariates were not included in the analysis (*F*(1, 72) = 6.04, *p* = .016, effect size = .075)

Table S9. Mean and standard deviations of proximity (distance in m) to avatar 1 and avatar 2 in each task stage in synchrony (*n* = 38) and non-synchrony (n = 38) conditions, and the results of the Mann-Whitney U tests.

|  |  | Avatar 1 | |  | | Avatar 2 | | |  |
| --- | --- | --- | --- | --- | --- | --- | --- | --- | --- |
| Distance (m) | Condition | Mean distance | SD | | *P* | | Mean distance | SD |  |
| **Overall average (across all stages)** | Synchrony | 1.395 | .080 | | .228 | | 1.404 | .071 | .280 |
|  | Non-Synchrony | 1.415 | .084 | |  |  | 1.391 | .081 |  |
| **Overall average change (corrected for start)** | Synchrony | -.002 | .025 | | .803 | | .001 | .032 | .811 |
|  | Non-Synchrony | -.001 | .014 | |  |  | .001 | .019 |  |
| **Start** | Synchrony | 1.396 | .077 | | N/A | | 1.404 | .063 | N/A |
|  | Non-Synchrony | 1.416 | .080 | |  |  | 1.390 | .083 |  |
| **Stage 1** | Synchrony | 1.394 | .080 | | N/A | | 1.403 | .071 | N/A |
|  | Non-Synchrony | 1.414 | .083 | |  |  | 1.390 | .081 |  |
| **Stage 2** | Synchrony | 1.396 | .080 | | N/A | | 1.406 | .072 | N/A |
|  | Non-Synchrony | 1.415 | .083 | |  |  | 1.392 | .082 |  |
| **Stage 3** | Synchrony | 1.396 | .082 | | N/A | | 1.407 | .071 | N/A |
|  | Non-Synchrony | 1.415 | .083 | |  |  | 1.391 | .082 |  |
| **Stage 4** | Synchrony | 1.392 | .082 | | N/A | | 1.402 | .072 | N/A |
|  | Non-Synchrony | 1.412 | .084 | |  |  | 1.388 | .081 |  |
| **Stage 5** | Synchrony | 1.394 | .081 | | N/A | | 1.405 | .072 | N/A |
|  | Non-Synchrony | 1.415 | .085 | |  |  | 1.391 | .082 |  |
| **Stage 6** | Synchrony | 1.394 | .081 | | N/A | | 1.405 | .072 | N/A |
|  | Non-Synchrony | 1.415 | .085 | |  |  | 1.392 | .083 |  |
| **Stage 7** | Synchrony | 1.392 | .082 | | N/A | | 1.402 | .071 | N/A |
|  | Non-Synchrony | 1.412 | .086 | |  |  | 1.389 | .082 |  |
| **Stage 8** | Synchrony | 1.395 | .081 | | N/A | | 1.405 | .072 | N/A |
|  | Non-Synchrony | 1.417 | .088 | |  |  | 1.392 | .083 |  |
| **Stage 9** | Synchrony | 1.395 | .082 | | N/A | | 1.405 | .074 | N/A |
|  | Non-Synchrony | 1.419 | .086 | |  |  | 1.394 | .083 |  |

## Supplementary Information 5

## Questionnaire questions, scales and translations

Note the terms ‘virtual human’, ‘virtual character’ and ‘avatar’ are used interchangeably throughout this document and other supplementary materials.

| Pre-activity questionnaire: | | | |
| --- | --- | --- | --- |
| **Item** | **Question (ENG)** | **Scale** | **Translation (SPANISH)** |
| Demographics | Age, Gender | N/A | *Porfavor introduce tu edad, sexo* |
| Mini-IPIP Personality scale | Extraversion: Am the life of the party; Do not talk a lot (R); Talk to a lot of different people at parties; Keep in the background (R); Agreeableness: Sympathize with others’ feelings; Am not interested in other people’s problems (R); Feel others’ emotions; Am not really interested in others (R); Conscientiousness: “Get chores done right away; Often forget to put things back in their proper place (R); Like order; Make a mess of things (R); Neuroticism: “Have frequent mood swings; Am relaxed most of the time (R); Get upset easily; Seldom feel blue (R); Intellect: Have a vivid imagination; Am not interested in abstract ideas (R); Have difficulty understanding abstract ideas; Do not have a good imagination (R) | 1 – 5 Likert Scale (Cooper, Smillie, & Corr, 2010). | *A continuación se presentan frases que describen los comportamientos de las personas. Por favor, utilizando la escala que encontrara a continuacion, indique su nivel de acuerdo con las siguientes frases como características de su forma de ser. Descríbete como eres en general y no como desearías ser en un futuro. Descríbete como te ves de forma honesta, en relación con otras personas que conoces de tu mismo sexo, y más o menos de tu misma edad. Por favor, lee cada frase cuidadosamente:*  *Soy el alma de la fiesta; Me compenetro con las emociones de los otros; Realizo mis tareas inmediatamente; Tengo cambios frecuentes de estado de ánimo; Tengo una imaginación fluida; No hablo mucho; No me interesan los problemas de las otras personas; A menudo olvido poner las cosas en su lugar; Estoy tranquilo la mayor parte del tiempo; No me interesan las ideas abstractas; En las festas hablo con diferentes tipos de personas; Me emocionan los sentimientos de los otros; Me agrada el orden; Me disgusto fácilmente; Tengo dificuldades para entender ideas abstractas; Intento no llamar la atención; No me intereso demasiado por los demás; Me hago un lío con las cosas; Raramente me siento triste; No tengo una buena imaginación* |
| Pre- and post-activity questions: | | | |
| **Item** | **Question (ENG)** | **Scale** | **Translation (SPANISH)** |
| Positive and Negative Affect Scale (PANAS) | Please indicate how you are feeling in this moment for determined, attentive, alert, inspired, active, afraid, nervous, upset, ashamed, and hostile | 1 – 5 Likert Scale where 1 = *not at all* and 5 = *extremely* (Thompson, 2007). | *Por favor indica cómo te sientes en este momento. Selecciona el opción que mejor describa tu respuesta.* Atento/a, Activo/a, Temeroso/a, Hostil, Avergonzado/a, Decidido/a, Molesto/a, Inspirado/a, Nerviso/a, Alerta |
| Post-training, pre-activity question: | | | |
| **Item** | **Question (ENG)** | **Scale** | **Translation (SPANISH)** |
| Confidence | How confident are you that you can remember these four dance moves and do them on demand? | 1 – 100 sliding scale, 1 = *very slightly* and 100 = *extremely* | *¿Qué tan seguro estás de que puedes recordar estos tres movimientos y hacerlos cuando se te pida?* |

| Post-activity questionnaire: | | | | | |
| --- | --- | --- | --- | --- | --- |
| **Item** | **Question (ENG)** | **Scale** | | | **Translation (SPANISH)** |
| Inclusion of Other in Self (IOS)^[[1]](#footnote-1)^* | *P*lease choose the picture that best describes your relationship with the other avatars in the virtual reality environment. | 1 – 7 pictorial scale with labelled circles of increasing overlap to indicate relationship between ‘*self*’ and ‘*group*’ (Aron, Aron, & Smollan, 1992) | | | *Porfavor escoge la imágen que mejor describa tu relación con los otros avatares del entrono virtual.* |
| Connectedness* | How connected did you feel to the other avatars? | 1 – 100 sliding scale where 1 = not at all and 100 = *very much*; adapted from (Wiltermuth & Heath, 2009) | | | *¿En qué medida te sentías conectado a los otros avatares?* |
| Likeability* | How likeable did the other avatars appear to you? | 1 – 100 sliding scale where 1 = *not at all* and 100 = *very much*; adapted from (Hove & Risen, 2009) | | | *¿Cómo de simpáticos te parecen los otros avatares?* |
| Similarity in personality* | To what extent do you feel similar in personality to the other avatars? | 1 – 100 sliding scale where 1 = *not at all* and 100 = *very much;* adapted from (Valdesolo & Desteno, 2011) | | | *¿En qué medida sientes que tu personalidad es similar a la de los demás avatares?* |
| Interest in meeting others | *How interested would you be to meet the other participants from your session?* | 1 – 100 sliding scale 1 = *not at all* and 100 = *very much* | | | *¿Cuán interesado estarias en conocer a los otros participantes de tu sesion de realidad virtual?* |
| Willingness to help | *If one of the other participants from your session needed help (e.g. to borrow 10 Euro), would you offer to help?* | 1 – 100 sliding scale where 1 = *not at all* and 100 = *very likely* | | | *Si uno de los participantes de tu sesión necesitará ayuda (p.ej. pedir prestado 10 euros), ¿le ofrecerías la ayuda?* |
| Willingness to receive help | *If you needed help (e.g. to borrow 10 Euro) and one of the other participants from your session offered to help (e.g. lend you 10 Euro), would you accept their help?* | 1 – 100 sliding scale where 1 = *not at all* and 100 = *very likely* | | | *Si tú necesitarás ayuda  (p.ej. pedir prestado 10 euros) y uno de los participantes de tu sesión se ofreciera a ayudarte (P.ej. prestándote 10 euros) ¿aceptarías su ayuda?* |
| Fun | How much fun did you have in the virtual reality environment? | 1 – 100 sliding scale where 1 = *not at all* and 100 = *very much* | | | *¿Cuánto te has divertido en el entorno virtual?* |
| Embarrassment | “How uncomfortable or embarrassed did you feel in the virtual reality environment?” | 1 – 100 sliding scale where 1 = *not at all* and 100 = *very much* | | | *¿Cómo de incómodo te has sentido en el entorno virtual?* |
| Difficulty | “How difficult were the movements?” | 1 – 100 sliding scale where 1 = *not at all* and 100 = *very much* | | | ¿Cuán difíciles te han parecido los movimientos? |
| Enjoyment | How enjoyable was the virtual reality task? | 1 – 100 sliding scale where 1 = *not at all* and 100 = *very much* | | | *¿Cuán agradable fue la tarea de realidad virtual?* |
| Success | In your opinion, how successful were you at performing the movements as instructed? | 1 – 100 sliding scale where 1 = *not successful* and 100 = *very successful* | | | *Según tu opinión, ¿cómo de exitosa ha sido la realización de los movimientos según las instrucciones?* |
| Synchrony | *How synchronized were your movements with the movements of the other avatars?* | 1 – 100 sliding scale where 1 = *not at all* and 100 = *extremely* | | | *¿En conjunto, cómo de sincronizados han sido tus movimientos con los movimientos de los otros avatares?* |
| Following others | *How often did you feel like you were following the movements of other avatars?* | 1 – 100 sliding scale where 1 = *never* and 100 = *always* | | | *¿Con qué frecuencia has sentido como si estuvieras siguiendo los movimientos de otros avatares?* |
| Being followed | *How often did you feel like the other avatars were following your movements?* | 1 – 100 sliding scale where 1 = *never* and 100 = *always* | | *¿Con qué frecuencia has sentido como si los otros avatares estuvieran siguiendo tus movimientos?* | |
|  | *How did you find the lighting in the virtual reality environment?* | 1 – 100 sliding scale where 1 = *too dark* and 100 = *too light* | | *¿Cómo has encontrado la iluminación en la experiencia de realidad virtual?* | |
| Real people | *To what extent did you feel that the other avatars were controlled by real people?* | 1 – 100 sliding scale where 1 = *not at all* and 100 = *very much* | | *¿En que grado sentiste que los otros avatares eran controlados por personas reales?* | |
| Feeling of being with other people | *In general, during the virtual reality experience, did you feel like you were with other people?* | 1 – 100 sliding scale where 1 = *not at all* and 100 = *very much* | | *¿En general, durante la experiencia virtual he tenido la sensacion de estar con otras personas?* | |
| Hypothesis check | What was the aim of this experiment? Please write down your best guess. | | N/A | *¿Cuál fue el objetivo de este experimento? Por favor, escribe debajo tu mejor valoración.* | |

##

## Instructions given to participants and translations

| Pre- training video instructions: | |
| --- | --- |
| **Instructions (ENG)** | **Translation (SPANISH)** |
| *You will now learn three basic movements. A video shown on the screen will explain each move slowly. Please join in with the video so that you can learn each move. Later you will join a group in the virtual reality environment. You will need to remember the name of each move, and how to do it. Please stand up and click next to begin the video.* | *Ahora aprenderás tres movimientos básicos. Un video te explicará cada movimiento. Por favor, haz cada movimiento a la vez que el video para aprenderlos. Después te unirás a un grupo en un entorno de Realidad Virtual dónde necesitarás recordar el nombre de cada movimiento y saber cómo se hace. Por favor, ponte en pie y haga clic en siguiente para empezar el video.* |
| Post- training video, pre-activity instructions: | |
| *You will now enter a virtual environment, where you and others have been randomly assigned avatars.* | *Ahora entrarás en un entorno virtual, donde a ti y a otros se os han asignados avatares al azar. Levántate para decirle al investigador que estas* |
| Post- training video, pre-activity instructions: | |
| *Welcome to the virtual reality environment where you will join with others to do a group movement task together.*  *You will have a short time now to get used to your avatar. Look to your left and you should see a reflection of your avatar.*  *<30 second embodiment time>*  *OK, the group movement task is about to start, you are now together with the others and you will join the group to do the movements you learnt earlier. Please turn so that you can see the other avatars.*  *The audio instructions will tell you what movement to do, and when to change to the next movement in the sequence.*  *Once you start with the first movement, keep moving the whole time – don’t stop!*  *Remember to keep repeating the movement in a smooth and continuous way, keeping at a relaxed, constant rhythm.*  *When you hear the instruction for the next movement, finish the movement you are currently doing and then smoothly transition to the next movement. Keep moving the whole time.*  *Now we are ready to begin to do the movements together. Please face the others and assume the start position, place your arms in a relaxed position on either side of your body.*  *The first movement is ‘V’, please begin now.*  *…‘Reach’…‘Cross’*  *Please finish this movement and return to the start position.*  *Well done the group movement task is now finished!*  *Please wait a minute while the researchers come to help remove your headgear.* | *Bienvenido al entorno de realidad virtual donde vas a realizar una tarea en grupo basada en movimientos conjuntamente con los demás.*  *Vas a disponer de un periodo de tiempo para acostumbrarte a tu avatar. Mira hacia tu izquierda y verás el reflejo de tu avatar.*  *<embodiment>*  *Vale, la tarea grupal de movimientos está a punto de empezar. Ahora estás con los demás y te unirás al grupo para realizar los movimientos que aprendiste antes. Por favor, vuélvete para ver los otros avatares.*  *Las instrucciones de audio te van a indicar qué movimiento hacer y cuándo cambiar al siguiente movimiento de la secuencia.*  *Una vez hayas empezado con el primer movimiento, sigue moviéndote todo el rato. ¡No te pares!*  *Recuerda repetir el mismo movimiento de una forma suave y continua, manteniendo un ritmo relajado y constante.*  *Cuando oigas la indicación del siguiente movimiento, primero acaba el movimiento que estés haciendo y luego pasa suavemente al siguiente movimiento. Recuerda que debes moverte todo el rato.*  *Ya estamos listos para empezar juntos con los movimientos. Por favor, ponte de cara a los demás y adopta la posición inicial, con los brazos relajados a ambos lados del cuerpo.*  *El primer movimiento es “V”, por favor, empieza ahora.*  *…'Alcanzar'… ‘Cruz’*  *Por favor, acaba tu movimiento y vuelve a la posición inicial.*  *Bien hecho! La tarea grupal de movimientos ha finalizado.*  *Por favor, espera un momento mientras el investigador viene a sacarte el equipo.* |

## Supplementary Information 6

## Movement training video <SI6_VR Movement Training Video.mp4>

## Supplementary Information 7

## Idle pose 1 <SI7_VR Idle pose 1.mp4>

## Supplementary Information 8

## Idle pose 2 <SI8_VR Idle pose 2.mp4>

## Supplementary Information 9

## 9.1 Latency defining file

The movement task was split into nine stages, with transition to the next stage marked by instruction to the participant to change to the next movement in the sequence. In each stage, the other two virtual characters (Character1 and Character2) were coded to delay their mirroring of the participants’ movements by a pre-coded latency. The delay differed between Character1 and Character2 to avoid these two characters being in synchrony with each other. Note that there are 60 frames in a second, so a delay of for e.g. 100 frames/second = delay of 1.66 seconds).

| ***Stage of movement task*** | ***Virtual character*** | ***Movement condition*** | ***Delay in frames/second*** |
| --- | --- | --- | --- |
| Stage1 | Character1 | NonSync | 100 |
| Stage1 | Character1 | Sync | 15 |
| Stage1 | Character2 | NonSync | 200 |
| Stage1 | Character2 | Sync | 35 |
| Stage2 | Character1 | NonSync | 115 |
| Stage2 | Character1 | Sync | 25 |
| Stage2 | Character2 | NonSync | 215 |
| Stage2 | Character2 | Sync | 28 |
| Stage3 | Character1 | NonSync | 130 |
| Stage3 | Character1 | Sync | 35 |
| Stage3 | Character2 | NonSync | 230 |
| Stage3 | Character2 | Sync | 20 |
| Stage4 | Character1 | NonSync | 145 |
| Stage4 | Character1 | Sync | 22 |
| Stage4 | Character2 | NonSync | 245 |
| Stage4 | Character2 | Sync | 15 |
| Stage5 | Character1 | NonSync | 160 |
| Stage5 | Character1 | Sync | 35 |
| Stage5 | Character2 | NonSync | 260 |
| Stage5 | Character2 | Sync | 20 |
| Stage6 | Character1 | NonSync | 145 |
| Stage6 | Character1 | Sync | 25 |
| Stage6 | Character2 | NonSync | 245 |
| Stage6 | Character2 | Sync | 35 |
| Stage7 | Character1 | NonSync | 130 |
| Stage7 | Character1 | Sync | 35 |
| Stage7 | Character2 | NonSync | 230 |
| Stage7 | Character2 | Sync | 25 |
| Stage8 | Character1 | NonSync | 115 |
| Stage8 | Character1 | Sync | 25 |
| Stage8 | Character2 | NonSync | 215 |
| Stage8 | Character2 | Sync | 20 |
| Stage9 | Character1 | NonSync | 110 |
| Stage9 | Character1 | Sync | 15 |
| Stage9 | Character2 | NonSync | 200 |
| Stage9 | Character2 | Sync | 10 |

**References:**

Aron, A., Aron, E. N., & Smollan, D. (1992). Inclusion of Other in the Self Scale and the Structure of Interpersonal Closeness. *Journal of Personality and Social Psychology*, *63*(4), 596–612.

Cooper, A. J., Smillie, L. D., & Corr, P. J. (2010). A confirmatory factor analysis of the Mini-IPIP five-factor model personality scale. *Personality and Individual Differences*, *48*(5), 688–91. http://doi.org/10.1016/j.paid.2010.01.004

Hove, M. J., & Risen, J. L. (2009). It’s all in the timing: Interpersonal synchrony increases affiliation. *Social Cognition*, *27*(6), 949–61.

Thompson, E. R. (2007). Development and validation of an internationally reliable short-form of the positive and negative affect schedule (PANAS). *Journal of Cross-Cultural Psychology*, *38*(2), 227–42. http://doi.org/10.1177/0022022106297301

Valdesolo, P., & Desteno, D. (2011). Synchrony and the social tuning of compassion. *Emotion*, *11*(2), 262–6.

Wiltermuth, S. S., & Heath, C. (2009). Synchrony and Cooperation. *Psychological Science*, *20*(1), 1–5.

1. * These 3 items were averaged to form the ‘Social Closeness Index’ [↑](#footnote-ref-1)
